# Scientific provision of an effective development of soybean breeding and seed production in the Russian Far East

**DOI:** 10.18699/VJ21.040

**Published:** 2021-07

**Authors:** V.T. Sinegovskaya

**Affiliations:** Federal Scientific Center “All-Russian Scientific Research Institute of Soybeans”, Blagoveschensk, Russia

**Keywords:** Russian Far East, soybean, cultivar, breeding and seed production, virus, fungal diseases, Дальний Восток, соя, сорт, селекция и семеноводство, вирусы, грибные болезни

## Abstract

In the Russian Far East, a highly profitable crop is soybean, which predominates in all farms’ crop rotation in the region. An increase in this crop production occurs here both by increasing the sown area and increasing its yield. Therefore, in scientific institutions, great attention is paid to breeding varieties that can produce high yields in conditions with limited thermal resources with adaptation to the extreme soil and climatic conditions of the region’s soybean growing zones. In 2020, 45 varieties developed by scientific institutions of the Far Eastern Federal District were introduced to the State Register of the Russian Federation and approved for use in production in code 12 region (Far Eastern), with the largest number of the entries coming from the All-Russian Scientific Research Institute of Soybeans. The share of cultivated areas in the Russian Far East occupied by domestic varieties was 63.7 %, the largest share of sown varieties – 48.9 % – belongs to the Federal Research Center All-Russian Scientific Research Institute of Soybean. The most popular were the varieties of the All-Russian Scientific Research Institute of Soybean, such as Alena, Kitrossa, Lydiya, Evgeniya, MK 100, Primorsky varieties (Musson, Primorskaya 4, Primorskaya 86, Primorskaya 96, Sphera) are in demand mainly in Primorsky Krai, and Khabarovsk varieties (Batya, Marinata) have an advantage in Khabarovsky Krai and the Jewish Autonomous Region. All varieties are not genetically modified and are created mainly by classical breeding methods. Breeders of the Federal State Budgetary Scientific Institution, “Federal Research Center of Agrobiotechnology of the Far East named after A.K. Chaika” and biotechnologists carry out the selection of pairs for crossing using biotechnological methods to assess their polymorphism, instead of long-term selection for phenotypic features in the field. Evaluation of domestic and foreign varieties for disease resistance revealed a high degree of damage to foreign varieties by dangerous viral and fungal diseases. Together with Japanese scientists from the University of Niigata, the astragalus mosaic virus was detected on Canadian and Chinese varieties in Primorsky Krai and the Amur Region using DNA analysis. The carrier of this disease is soybean aphid (Aphis glycines).

## Abstract (ru)

На Дальнем Востоке соя – высокорентабельная культура, ее посевы преобладают в севообороте всех хозяйств региона. Наращивание производства зерна этой культуры происходит за счет увеличения посевных площадей и повышения ее урожайности. Поэтому в научных учреждениях региона огромное внимание уделяется селекции сортов, обладающих способностью в условиях с ограниченными тепловыми ресурсами давать высокие урожаи. В статье приведены основные результаты, полученные в учреждениях Дальневосточного федерального округа (ДФО) по селекции сои. В 2020 г. 45 сортов сои, созданных научными учреждениями ДФО, зарегистрированы в «Государственном реестре селекционных достижений…» и допущены к использованию в производстве по 12-му (Дальневосточному) региону. Большинство из этих сортов принадлежит Всероссийскому научно-исследовательскому институту сои (ВНИИ сои). На долю посевных площадей сои Дальнего Востока, занятых сортами отечественной селекции, приходится 63.7 %, при этом 48.9 % составляют сорта ВНИИ сои. Самыми востребованными являются сорта ВНИИ сои, такие как Алена, Китросса, Лидия, Евгения, МК 100. Сорта приморской селекции – Муссон, Приморская 4, Приморская 86, Приморская 96, Сфера – пользуются спросом в основном в Приморском крае, а сорта хабаровской селекции – Батя, Марината – имеют преимущество в Хабаровском крае и Еврейской автономной области. Все сорта генетически не модифицированы и создаются главным образом классическими методами селекции. Селекционеры ФГБНУ «ФНЦ агробиотехнологий Дальнего Востока им. А.К. Чайки» совместно с биотехнологами проводят подбор пар для скрещивания с применением биотехнологических методов по оценке их полиморфизма вместо многолетнего отбора по фенотипическим признакам в полевых условиях. Оценка отечественных и зарубежных сортов на устойчивость к болезням позволила выявить высокую степень поражения зарубежных сортов опасными вирусами и патогенными грибами. Совместно с японскими учеными из университета Ниигата обнаружен вирус карликовости астрагала (MDV, Milk vetch dwarf virus) на канадских и китайских сортах в Приморском крае и Амурской области. Переносчик этого заболевания – соевая тля (Aphis glycines).

## Introduction

**Fig. 1.**
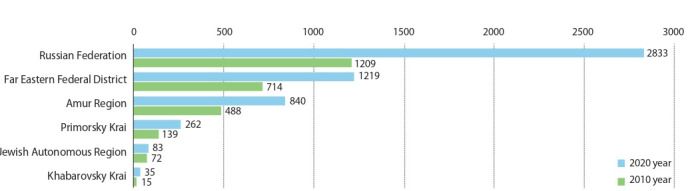
The sown area of soybean in Russia (thousand ha) in 2020 and 2010 years.

Soybean (Glycine max (L.) Merrill) as a valuable protein and oilseed crop plays a strategic role in the economies of many countries. Over the past decade, it has the highest production growth rates (Sinegovskii, Kuzmin, 2020). At present, Russia ranks 7th in world production with a sowing area of about 3.0 million hectares (Fig. 1). In world production, the 1st place belongs to Brazil – 36.9 million hectares (30 % of the global area), the second – the USA– 30.4 million hectares (25 %), the third – Argentina – 17.5 million hectares (14 %).

In recent years, soybean production in Russia has shown a stable positive trend (Malashonok, 2018; Dorokhov et al., 2019; Rasulova, Melnik, 2020). The increase in sown areas in 2020 compared to 2010 amounted to 134 %, and gross production increased by 279 %. The main regions of soybean cultivation in Russia are the Amur Region, Primorsky Krai, the Kursk and Belgorod Regions, Krasnodarsky Krai, which account for 62 % of all sown areas. The share of this crop in the Far East is 44 % of the total Russian (Sinegovskii, 2020). Soybean production is growing not only due to an increase in acreage but also due to an increase in crop yields, which is ensured by an increase in the potential productivity of new varieties (Sinegovskaya, Fokina, 2018; Butovets, Strashnenko, 2020). 

## The results of soybean breeding research

Three scientific institutions carry out scientific support of the soybean industry in the Far Eas: Federal Research Center All-Russian Scientific Research Institute of Soybean (Blagoveshchensk), Federal Research Center of Agrobiotechnology of the Far East named after A.K. Chaika” (Ussuriysk, Primorskiy Krai) and Far Eastern Agricultural Research Institute (Vostochnoye village, Khabarovsk Krai). The main direction of scientific work in all scientific institutions is the creation of varieties adapted to the Far East’s extreme conditions and resistant to the main harmful organisms, the production of original seeds and the development of innovative methods of their cultivation (Table 1). 

**Table 1.**
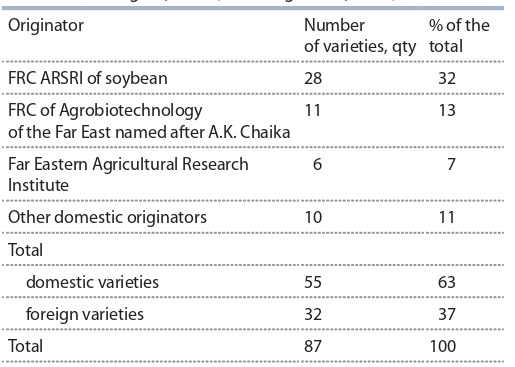
Soybean varieties approved for use in the Far East region, 2020 (State Register…, 2020)

**Fig. 2.**
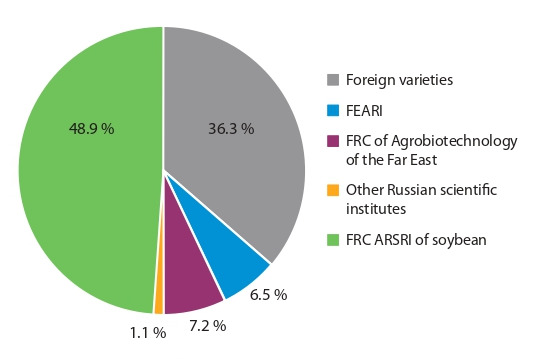
Share of soybean varieties (%) used in production in the Far Eastern Federal District, 2020

In 2020, the “State Register of Selection Achievements Authorized for Use for Production Purposes” of the Russian Federation contained 45 varieties of selection of the scientific institutions of the Far East, approved for use in production in 12 regions, the largest number of which belongs to the ARSRIS (State Register…, 2020). The share of sown areas in the Far East, occupied by varieties of domestic selection, was 63.7 %, the largest share of sown varieties belongs to the ARSRI of soybean – 48.9 % (Fig. 2). In Primorsky Krai, varieties of FRC of Agrobiotechnology of the Far East accounted for 7.2 %, and varieties of FEARI accounted for 6.5 %. Varieties of foreign selection occupied 36.3 % of all sown areas of the Far Eastern Federal District.

In general, in the Far East in 2020, 78 varieties of soybeans of domestic and foreign varieties were used for sowing, of which 19 varieties were of the breeding of ARSRI of soybean, occupying an area of sowing of 484.9 thousand hectares, three varieties – Far Eastern Agricultural Research Institute with a sowing area of 64.9 thousand hectares, ten varieties – FRC of Agrobiotechnology of the Far East, cultivated on an area of 72.0 thousand hectares. The total sowing area of domestic varieties of Far Eastern varieties was 621.8 thousand hectares, foreign varieties – 358.7 thousand hectares. The most popular were the varieties of the ARSRI of soybeans, such as Alena, Kitrossa, Lydiya, Evgeniya, MK 100, and others. In 2019, a new early ripening variety Sentyabrinka was included in the “State Register …” (2019), and already this year, at the request of farms, the institute produced 32 tons of original seeds of this variety, which is in demand by commodity producers as a high-yielding (3.0 t/ ha) with a protein content of more than 40 %. Primorsky breeding varieties: Musson, Primorskaya 4, Primorskaya 86, Primorskaya 96, Sphera are in demand mainly in Primorsky Krai. Varieties of the Khabarovsk breeding Batya, Marinata are sown in the Khabarovsk Territory and the Jewish Autonomous Region. This year, a new soybean variety, Khabarovsky yubilyar, is included in the “State Register ...” (2020), and 0.8 tons of original seeds have already been grown for commodity producers (Table 2).

**Table 2.**
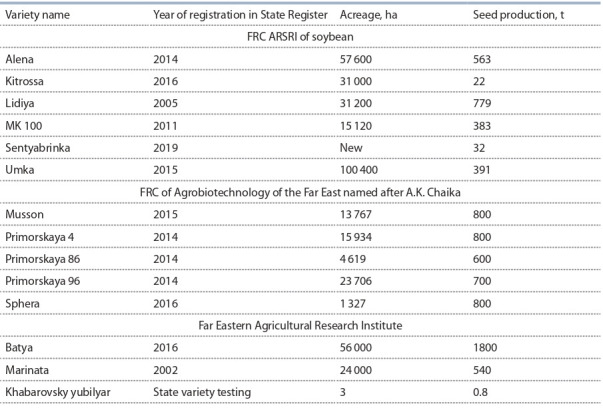
Production of original soybean seeds by scientific institutions of the Far Eastern Federal District, 2020

The protein content and yield of soybean seeds depending on a variety 

**Fig. 3.**
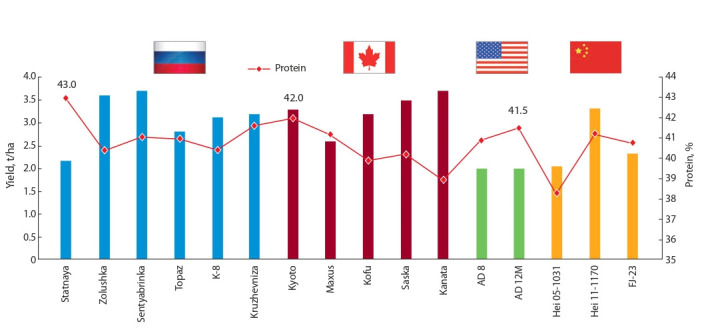
Yield and protein content in seeds of soybean varieties of various genetic origin, the average for 2018–2019

In solving the country’s food security, the size and the quality of the crop are important. In this direction, the institutes are working on creating soybean varieties with high protein content in seeds (Fig. 3).

Evaluation of domestic and foreign varieties grown on the experimental field of the ARSRI of soybean in identical conditions in terms of the protein content in seeds and the value of yield showed that Far Eastern varieties are not only not inferior to Canadian, Chinese, and American varieties, but surpass them both in seed quality and terms of yield (see Fig. 3) (Kodirova et al., 2020; Sinegovskaya et al., 2020). Foreign varieties, generally, have a long growing season that exceeds the frost-free period of the cultivation region, and producers receive soybeans damaged by frost. Foreign varieties showed an adverse reaction to the length of the day, temperature regime, waterlogging of the soil during pod formation, which is confirmed by the high abortion rate of pod ovaries, a low number of seeds in pods, and a decrease in plant productivity. The varieties of Russian breeding, having a shorter growing season, have time to ripen in a short frost-free period and are resistant to the main diseases and pests of soybeans (Vasina et al., 2019; Butovets, Strashnenko, 2020).

In 2020, the yield of mid-ripening Amur varieties varied from 2.12 to 3.43 t/ha, and Chinese – from 2.03 to 3.32 t/ha. The yield of Canadian and American varieties was 0.19…0.63 t/ha less than the varieties bred by the ARSRI of soybean.

Assessment of soybean varieties for disease resistance

Far Eastern soybean varieties have advantages in disease resistance over foreign, mainly Canadian and Chinese varieties, widely advertised in the Far East and imported for sale to producers of the region (Barsukova et al., 2015; Vasina et al., 2019). 

**Fig. 4.**
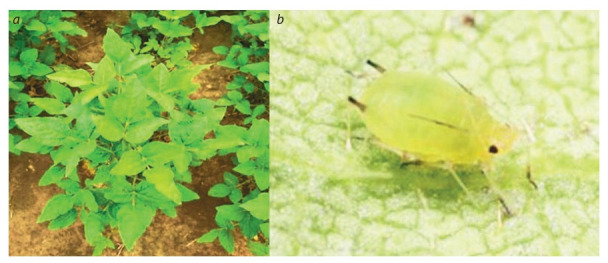
Soybean plants infected with the astragalus dwarf virus (Milk vetch dwarf virus) (a); the virus carrier is soybean aphid (Aphis glycines Matsumura) (b).

Evaluation of domestic and foreign varieties revealed a high degree of damage to Canadian and Chinese varieties by dangerous viruses and pathogenic fungi. In cooperation with Japanese scientists from Niigata University, using DNA markers, the astragalus dwarf virus (MDV, Milk vetch dwarf virus) was detected on Canadian and Chinese varieties in Primorsky Krai and the Amur Region. The carrier of this disease is aphids (Fig. 4).

The cultivars of the Amur breeding showed resistance to the development of this viral disease. The Canadian variety Maxus was affected by the virus mosaic of soybean (Soybean mosaic potyvirus) by the stage of seed filling up to 25 %, and the sample of the Chinese variety – by 50 %, which indicates weak resistance and danger for infection of other soybean varieties growing nearby. The virus causes leaf chlorosis and plant dwarfism.

**Fig. 5.**
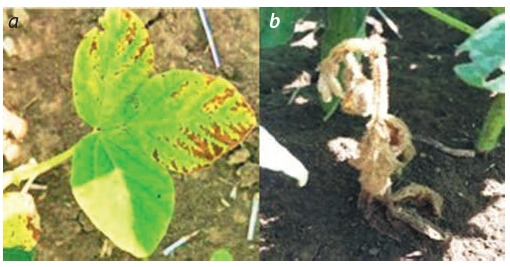
Damage to leaves (a) and plants (b) of soybeans with bacterial wilt.

During the research, for the first time bacterial wilt (Curtobacterium f laccumfaciens pv. f laccumfaciens (Hedges) Dowson) was discovered on American, Canadian and Chinese varieties, leading to the wilting of the plant and its further death (Fig. 5). The degree of infection with bacterial wilt (C. f laccumfaciens pv. f laccumfaciens) has not yet exceeded the harm threshold and amounted to 10 %, but the further spread of this bacterial disease can lead to significant death of soybean crops.

Severe disturbances in crop rotation in the Far East region led to the spread of soybean cyst nematode (Heterodera glycines Ichinohe). Inspection of the Amur Region fields for the presence of this pest has revealed lesions of the root system. Evaluation of our soybean varieties for resistance to nematodes artificially infected showed that the root system of plants of Sentyabrinka, Evgeniya, Sonata and Kukhanna varieties was completely free of the pest by the phase of full pods.

Far Eastern varieties are also resistant to fungal diseases such as Peronospora sparsa (Peronospora manshurica Naum.), Cercospora (Cercospora sojina Hara.), Phyllosticta (Phyllosticta sojaecola Massal.), Cercospora blight (Cercospora kikuchii (Matsuet Tomoyasu) Yardn.) and Septoria (Septoria glycines Hemmi.). Work on creating varieties resistant to fungal diseases is carried out annually using quarantine areas, based on studying the physiological and biological characteristics of varieties. All varieties are not genetically modified, which attracts the attention of the countries of the Asia-Pacific region (China, Korea, Japan). 

## The results of fundamental research in soybean breeding

In recent years, fundamental research in soybean breeding has been significantly strengthened in the region, which is ensured by the interaction of joint works in physiology, biotechnology, and genetics. Under the national project “Science”, in order to deepen fundamental research in 2019, two new laboratories were created and are operating at the ARSRI of Soybean, the laboratories of biotechnology and plant physiology, at the FRC of Agrobiotechnologies of the Far East – the laboratory for breeding and genetic research of field crops and Far Eastern Agricultural Research Institute – the laboratory of breeding cereals and legumes

**Fig. 6.**
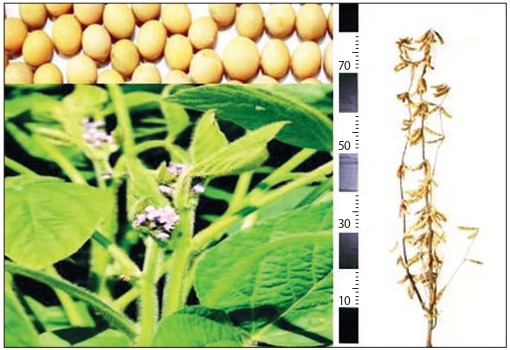
Early maturing soybean variety Luchistaya

A multidisciplinary approach, including knowledge of genetics, biochemistry, physiology, and plant breeding, makes it possible to create varieties with a wide range of phenotypic plasticity and resistance to external unfavorable environmental factors (Koshkin, 2010; RahimzadehBajgiran et al., 2012; Shcherban, 2019). In the ARSRI of Soybean, in a long-term study of the genetic collection of soybeans, physiologists isolated varieties with a high level of assimilation of the photosynthetically active part of the daylight spectrum, which they passed on to breeders for inclusion in the breeding process (Sinegovskaya, Tolmachev, 2011; Sinegovskaya, Dushko, 2017). The joint work of physiologists and breeders created a new soybean variety with a high level of absorption of photosyn hetically active light quanta. The new variety, called Luchistaya, has early maturity, exceeds the yield standard by 0.33 t/ha, with a productivity potential of 3.12 t/ha (Fig. 6). The variety belongs to the Manchurian subspecies (Glycine max ssp. manshurica (Enken) Zel. et Koch.), the approbation group – f lavida Enk, the growing season is 105–107 days, the protein content is 39.8–40.7 %.

An indeterminate type of growth characterizes the variety, the stem is straight, forms 2–4 long and short branches. The height of the plants is 72–85 cm, the height of attachment of the lower pods is 14 cm, the leaf is pointed ovoid, the flower is purple. Seeds are yellow, spherical, hilum is seed-colored and oval-shaped. The mass of 1000 seeds is 124.8–148.8 g. The variety is resistant to common pathogens, waterlogging and lodging, it is characterized by the increased photosynthetic activity of the leaf apparatus.

The creation of a new variety of classical breeding methods requires 15–20 years. To reduce the time of breeding new highly productive varieties, the breeders of the FRC of Agrobiotechnology of the Far East named after A.K. Chaika together with biotechnologists select pairs for crossing using biotechnological methods based on the assessment of their polymorphism instead of longterm selection for phenotypic traits in the field (Fisenko, Butovets, 2019).

## Conclusion

Scientific institutions of the Far East create highly productive soybean varieties, under production conditions, which are capable of providing soybean yield in the region of at least 2.5 t/ha, therefore the share of domestic varieties in soybean crops remains at a stable level and should increase, which requires an increase in the production of original seeds of new varieties and an improvement of their quality. The advantages of the varieties bred by the scientific institutions of the Far East are confirmed by their resistance to major diseases and harmful organisms compared to foreign varieties. The involvement in the breeding process of biotechnology methods and the study of physiological processes in photosynthesis is already yielding positive results and is the key to creating highly productive and high-quality varieties of a new generation. Besides, the low rates of renewal of the outdated material and technical base, as well as the lack of instrumentation of the regional scientific institutions engaged in the breeding and seed production of the strategic crop – soybean – restrain the rate of increase in this production of valuable high-protein crop. 

## Conflict of interest

The authors declare no conflict of interest.

## References

Barsukova E.N., Efremova O.S., Romashova M.V., Fisenko P.V.,
Ilyushko M.V. Efficiency of using biotechnological methods in
breeding of agricultural plants in Primorsky Scientific Research
Institute of Agriculture. Agrarnaya Rossiya = Agrarian Russia.
2015;8:2-7. (in Russian)

Butovets E.S., Strashnenko T.N. Study of soy varieties bred in the
Primorsky Krai, Russian Far East. Agrarnyy Vestnik Primor’ya =
Primorye Agrarian Bulletin. 2020;3(19):10-13. (in Russian)

Dorokhov A.S., Belyshkina M.E., Bolsheva K.K. Soy production in
the Russian Federation: basic trends and development prospects.
Vestnik Ulyanovskoy Gosudarstvennoy Selskohosyaistvennoy
Akademii = Bulletin of Ulyanovsk State Agricultural Academy.
2019;3(47):25-33. DOI 10.18286/1816-4501-2019-3-25-33. (in
Russian)

Fisenko P.V., Butovets E.S. Molecular-genetic approaches in soybean at A.K. Chaika Federal Scientific Center Agrobiotechnology of the Far East. Plodovodstvo i Yagodovodstvo Rossii = Pomiculture and Small Fruits Culture in Russia. 2019;59:343-353.
DOI 10.31676/2073-4948-2019-59-343-353. (in Russian)

Kodirova G.A., Kubankova G.V., Nizkiy S.E., Fisenko P.V. Evaluation of protein content in seed material of somaclonal soybean lines. Dalnevostochniy Agrarniy Vestnik = Far Eastern
Agrarian Bulletin. 2020;3(55):41-47. DOI 10.24411/1999-6837-
2020-13032. (in Russian)

Koshkin E.I. Physiology of Crop Resistance. Moscow, 2010. (in
Russian)

Malashonok A.A. Soybean production and use in the Russian
Federation. Teoreticheskiye i Prikladniye Problemy Agropromyshlennogo Kompleksa = Theoretical and Applied Problems
of Agro-industry. 2018;5(38):60-64. DOI 10.32935/2221-7312-
2018-38-5-60-64. (in Russian)

Rahimzadeh-Bajgiran P., Munehiro M., Omasa K. Relationships
between the photochemical reflectance index (pri) and chlorophyll fluorescence parameters and plant pigment indices at different leaf growth stages. Photosynth. Res. 2012;113:261-271.
DOI 10.1007/s11120-012-9747-4.

Rasulova V.A., Melnik A.F. Analysis of the current state of soybean production in Russia. Vestnik Selskogo Razvitiya i Sotsialnoy Politiki = Bulletin of Agricultural Development and Social
Policy. 2020;3(27):6-8. (in Russian)

Shcherban A.B. HD-Zip genes and their role in plant adaptation
to environmental factors. Russ. J. Genet. 2019;55(1):1-9. DOI
10.1134/S1022795419010125.

Sinegovskaya V.T., Dushko O.S. Investigation of photosynthetic
processes in soybean varieties bred in the Amur region. Vestnik
Rossiyskoy Selskokhozyaystvennoy Nauki = Bulletin of Russian
Agricultural Science. 2017;3:54-56. (in Russian)

Sinegovskaya V.T., Fokina E.M. Soybean selection as an instrument for solving import substitution tasks in the far eastern federal district. Trudy Kubanskogo Gosudarstvennogo Agrarnogo
Universiteta = Proceedings of the Kuban State Agrarian University. 2018;72:328-331. DOI 10.21515/1999-1703-72-328-331.
(in Russian)

Sinegovskaya V.T., Ochkurova V.V., Sinegovskii M.O. Content of
protein and fat in soybean seeds of various genetic origin. Rossiyskaya Selskokhozyaystvennaya Nauka = Russian Agricultural
Sciences. 2020;5:15-19. DOI 10.31857/S250026272005004X.
(in Russian)

Sinegovskaya V.T., Tolmachev M.V. Evaluation of soybean varieties according to photosynthetic activity in the Amur region. In:
Proceedings of the International Scientific-Practical Conference
“Agricultural Problems of the Soybean Sowing Territories of
Asia and Pacific”. 2011;46-50. (in Russian)

Sinegovskii M.O. Perspectives of soybean production in the Far
East federal district. Vestnik Rossiyskoy Selskokhozyaystvennoy
Nauki = Bulletin of Russian Agricultural Science. 2020;1:13-16.
DOI 10.30850/vrsn/2020/1/13-16. (in Russian)

Sinegovskii M.O., Kuzmin A.A. State, prospects and phytosanitary risks of soybean production. Zashchita i Karantin Rasteniy = Plant Protection and Quarantine. 2020;10:7-11. DOI
10.47528/1026-8634_2020_10_7. (in Russian)

State Register of Selection Achievements Authorized for Use
for Production Purposes. Vol. 1. Plant Varieties (official publication). Moscow: Rosinformagrotekh Publ., 2019. (in Russian)

State Register of Selection Achievements Authorized for Use
for Production Purposes. Vol. 1. Plant Varieties (official publication). Moscow: Rosinformagrotekh Publ., 2020. (in Russian)

Vasina E.A., Butovets E.S., Dega L.A. Distribution of soybean varieties on resistance to pathogens in environmental geographic
groups. Zashchita i Karantin Rasteniy = Plant Protection and
Quarantine. 2019;6:50-52. (in Russian)

